# Are We Degenerating?

**Published:** 1894-10

**Authors:** 


### Editorial.

### ARE WE DEGENERATING?

The professional wail that dentistry in America is losing ground, and is no better and probably not as far advanced as it was forty years ago, has been sounded in two conventions the past mid-summer,—the Section of Oral and Dental Surgery, American Medical Association, and the Mid-winter Fair Dental Congress, both meeting in California.

While we are in general sympathy with the trend of the remarks made there, and must regard with favor every effort to infuse more activity into the work of our profession, we cannot endorse the wholesale denunciation there made, or the hypercriticism that seemed to prevail in much of the discussion. If these could be sustained upon a careful analysis of the work performed in the past forty years, it might be proper to write degenerated on our outposts for all the outside world to accept as the estimate we place upon ourselves.

There is such a thing as leading without destroying; infusing higher ambitions without undermining the foundations on which a superstructure has been built; but this seems to have been mainly lost sight of in the consideration of the question.

It is unfortunately too true that much that has been written for conventions and journals is a "rehash" of old work, and that many of the papers are the preparation of a day, or a week, and have no real value except they serve to give temporary notoriety to the individuals preparing them. It is also equally true that science does not deal in such material, but endeavors to seek truth at the fountain-head by laborious experiments and, it may be, years of investigation. While this is admitted as true of dentistry in America, as it is of dentistry everywhere, it is equally so of all other professions.

"If we wish our profession to stand high, . . . we should follow the lead of the regular medical profession." This was the counsel given by one of the essayists, and he, doubtless, honestly believed it to be the only course to pursue. It is true that the few in medicine have struggled through the centuries to make it a scientific profession, and have accomplished much in that direction, but it is lamentably the fact that in its practice it falls very far short of that scientific exactness that dentistry has secured in its half-century of work. In fact, we think it can be truthfully said that whatever of science has been secured to medicine has been accomplished by specialists. How many years has it been since specialism in the profession of medicine was regarded with extreme disfavor? Twenty-five years ago the man who dared to step beyond the general practice was regarded with almost the contempt that was vouchsafed to the dentist of the period. All this is now changed, and the specialist has become the leader, the scientist of the medical profession, the one, in fact, to whom the general practitioner must look up to for guidance. Dentistry led, by force of circumstances, the line of special workers. Its claims were treated with derision, and its efforts to broaden culture as the vain attempts of a race of mechanics to bring their work up to a professional standard.

For one hundred and fifty years, and more, perhaps, the French, English, and subsequently the American dentists have labored incessantly to build a profession on scientific principles. At no period in all that time have the representative men in dentistry failed in their duty. Every step has been laboriously won, and but for this faithfulness those who so seriously attacked its worth in the California conventions would not have been able to attend there as delegates, nor would these meetings have been a possibility. The work has been unceasing and not confined to any one country; each of the civilized nationalities has had an honorable share in the labor. We are not disposed to overestimate the part taken by America in this advance, but it has added no inconsiderable portion to the scientific wealth of the world's dental knowledge.

It is in bad taste, in our opinion, to single out a large body of men for special denunciation, and to enforce this by comparing the results obtained with the work of other people. We question the truth of the statement that "our transatlantic *confrères* produce an aggregate of scientific work in dentistry which far exceeds the output in this country." In a somewhat extended acquaintance with foreign dental literature we have failed to discover the correctness of this, but there has been recognized a painstaking effort in contributions to give, what may be truthfully said to be rare in the United States, full quotations and due credit for previous discoveries in the same line of work.

It is unfortunately too true that the laborious scientist in this country has had in the past no recognition. The egotistic assumption of the arrogant German, quoted at the meeting, that an important discovery in medicine by an American had been overlooked because "medical science (in America) had *been regarded as still in its kinderschule*," illustrates exactly how difficult it has been for an American to make an impression. We have no desire to be personal in this article, but it may be said with truth that had the distinguished discoverer of the cause of dental caries produced the same result in America, he would not have had an audience; but, having been fortunately located in Germany, he had the entire world to recognize the correctness of his claims and the thoroughness of his investigation.

It is discreditable to this country that this should be. It is a time for self-assertion, and not self-depreciation. The assumptions derogatory to the work performed here, prevalent in this country and abroad, should be met with a dignified spirit of denial. We are not prepared to believe it does exist to any extent in foreign countries, but it would not be surprising if it became the general opinion, if many more such convocations discussed the subject as was done in California.

True science can never have many close followers in any profession. It means a self-sacrificing life; comparative poverty follows in its train, and the only hope of reward must be found in the pleasure of the work and the consciousness that truth has been exalted by the efforts. Such unselfishness must always be rare.

Dentistry is peculiar in that the individual must be remarkably constituted to be able to devote himself to scientific work and make money by his profession. The two things do not harmonize. Hence the number of scientists in it must be limited, for the practice is too exhausting to admit of extended devotion to it and laborious investigation in untrodden fields. This is also largely true of medicine, hence we find in that profession that those who devote themselves to original work are rarely practitioners in the accepted sense.

Dentistry has accomplished a great work in a half-century. In some things the advance has been of a most brilliant character, and along all lines the higher education prevailing in all our best schools is sending out men who will not always be practitioners in the moneyed sense, but who will make a name on the roll of fame worthy to stand with any of the world's people. It stands to-day equal to any of the specialties of medicine, and this has been accomplished by a steady and persistent advance in scientific work. While we may wish for better things in the future, let us not deprecate the good accomplished, or lessen our self-respect by unjust comparisons.

